# Information Geometry of Nonlinear Stochastic Systems

**DOI:** 10.3390/e20080550

**Published:** 2018-07-25

**Authors:** Rainer Hollerbach, Donovan Dimanche, Eun-jin Kim

**Affiliations:** 1 Department of Applied Mathematics, University of Leeds, Leeds LS2 9JT, UK; 2 School of Mathematics and Statistics, University of Sheffield, Sheffield S3 7RH, UK; 3 Institut National des Sciences Appliquées de Rouen, 76801 Saint-Étienne-du-Rouvray CEDEX, France

**Keywords:** stochastic processes, Fokker-Planck equation, information length

## Abstract

We elucidate the effect of different deterministic nonlinear forces on geometric structure of stochastic processes by investigating the transient relaxation of initial PDFs of a stochastic variable *x* under forces proportional to $-x^{n}$ (n=3,5,7) and different strength *D* of δ-correlated stochastic noise. We identify the three main stages consisting of nondiffusive evolution, quasi-linear Gaussian evolution and settling into stationary PDFs. The strength of stochastic noise is shown to play a crucial role in determining these timescales as well as the peak amplitude and width of PDFs. From time-evolution of PDFs, we compute the rate of information change for a given initial PDF and uniquely determine the information length L(t) as a function of time that represents the number of different statistical states that a system evolves through in time. We identify a robust geodesic (where the information changes at a constant rate) in the initial stage, and map out geometric structure of an attractor as $L\left(t\to \infty \right)\propto \mu^{m}$, where μ is the position of an initial Gaussian PDF. The scaling exponent *m* increases with *n*, and also varies with *D* (although to a lesser extent). Our results highlight ubiquitous power-laws and multi-scalings of information geometry due to nonlinear interaction.

## 1. Introduction

There is increasing interest in a metric on probability from theoretical and practical considerations, with different metrics proposed depending on the question of interest (e.g., [1,2,3,4,5,6,7,8,9,10] and further references therein). Theoretically, the assignment of an appropriate metric to probability enables us to mathematically quantify the difference among different Probability Density Functions (PDFs), providing a beautiful conceptual link between a stochastic process and geometry. At a practical level, it can be utilized for optimising various desired outcomes. For instance, the Wasserstein metric has been extensively studied for the optimal transport problem to minimize transport cost, typically taken to increase quadratically with distance between two locations [1]. The Fisher (also called Fisher-Rao) metric has recently been used for optimization, including the minimization of entropy production [11], parameter estimation [12], controlling population [13], understanding the arrow of time [14], and analysing the convexity of the relative entropy [15].

However, compared with the Wasserstein metric, whose application has established itself as a branch of applied mathematics, the geometric structure associated with the information change in the Fisher metric and its utility have been explored much less. Unlike the Wasserstein distance, the Fisher metric yields a hyperbolic geometry in the upper half-plane (e.g., [9,10]) where the distance is measured in units of the width of the PDF. That is, the distance in the Fisher metric is dimensionless and represents the number of different statistical states. Consequently, for a Gaussian PDF, statistically distinguishable states are determined by the standard deviation; two PDFs that have the same standard deviation and differ in peak positions by less than one standard deviation are statistically indistinguishable (e.g., [11,16,17,18,19,20,21,22]).

By extending the Fisher metric to time-dependent problems, we recently introduced a system-independent way of quantifying information change associated with time-evolution of PDFs [13,23,24,25,26,27,28,29,30]. (Note that we use information for statistically different states, refraining from the debate on the exact definition of information, e.g., [5,31].) The key idea is to define an infinitesimal distance at any time by comparing two PDFs at adjacent times and sum these distances. The total distance gives us the number of statistically different states that a system passes through in time, and is called information length L. While the detailed derivation of L is given in [5,13,16,23,24,25,26,27,28,29,30], it is useful to highlight that L is a measure of the total elapsed time in units of a dynamical timescale for information change. To show this, we define the dynamical time τ(t) as follows:

(1) $$E\equiv \frac{1}{{\left[\tau \left(t\right)\right]}^{2}}=\int \frac{1}{p(x,t)}{\left[\frac{\partial p(x,t)}{\partial t}\right]}^{2}\;dx.$$

Here, τ(t) is the characteristic timescale over which the information changes. Having units of time, τ(t) quantifies the correlation time of a PDF. Alternatively, 1/τ quantifies the (average) rate of change of information in time. A particular path which gives a constant valued E is a geodesic along which the information propagates at the same speed [13]. L(t) is then defined by measuring the total elapsed time *t* in units of τ as:

(2) $$L\left(t\right)=\int_{0}^{t}\frac{dt_{1}}{\tau (t_{1})}=\int_{0}^{t}\sqrt{\int dx\frac{1}{p(x,t_{1})}{\left[\frac{\partial p(x,t_{1})}{\partial t_{1}}\right]}^{2}}\;dt_{1}.$$

L(t) is a Lagrangian quantity (unlike entropy or relative entropy), uniquely defined as a function of time *t* for a given initial PDF, and represents the total number of statistically distinguishable states that a system evolves through. It thus provides a very convenient methodology for measuring the distance between p(x,t) and p(x,0) continuously in time for a given p(x,0). One of the utilities of L(t) is to quantify the proximity of any initial PDF to a final attractor of a dynamical system. We note that for a linear process, we can express the information length in terms of a metric tensor $g_{ij}$ using the two parameters $\beta =1/2\langle {\left(x-\langle x\rangle \right)}^{2}\rangle$ and 〈x〉 (e.g., [13]). However, even in the case where the control parameters are not known, we can still define L as long as we can compute time-dependent PDFs (e.g., from data [24]).

Traditionally, concepts such as bifurcations, Lyapunov exponent or a distance to an equilibrium point are commonly used to understand dynamical systems (e.g., see [32]). An intriguing question arises as to how to define a distance between a point, say *x*, and an equilibrium which is not a point but a limit cycle, or chaotic attractor. One interesting possibility is to consider a narrow initial PDF around *x* and to measure the total information length $L\left(t\to \infty \right)=L_{\infty }$ as the initial PDF evolves toward the equilibrium PDF. $L_{\infty }$ offers a Lagrangian distance that depends on the trajectory/history of the system (e.g., time-dependent PDF), being uniquely defined as a function of time for a given initial PDF. This enables us to map out the attractor structure by measuring $L_{\infty }$ for different locations of a narrow initial PDF. In a chaotic system, $L_{\infty }$ changes abruptly when a different initial PDF is used, as shown in Figure 4 in [24], where the very spiky curve represents a sensitive dependence of $L_{\infty }$ on x(t=0), the location of a very narrow initial PDF. This sensitive dependence of $L_{\infty }$ on x(t=0) means that a small change in the initial condition causes a large difference in a path that a system evolves through and thus $L_{\infty }$. This is quite similar to the sensitive dependence of the Lyapunov exponent on the initial condition. That is, our L provides a new methodology to test chaos. Furthermore, Figure 4 in [24] shows small $L_{\infty }$ for unstable points, demonstrating that unstable points are more similar to chaotic attractors.

The purpose of this paper is to consider the effect of different orders of nonlinear interaction on the geometric structure by considering the case when the equilibrium is a stable point [26,33]. The remainder of this paper is organised as follows: Section 2 introduces the basic model. Section 3 derives exact analytic solutions in the absence of stochastic noise, as well as asymptotic scalings for the timescales, peak amplitudes and widths once noise plays a role. Section 4 presents numerical results, and shows how they compare with the analytic scalings. Section 5 summarizes the results.

## 2. Model

We consider the following nonlinear Langevin equation:

(3) $$\frac{dx}{dt}=-\gamma x^{n}+\xi .$$

Here, *x* is a random variable and ξ is a stochastic forcing, which for simplicity can be taken as a short-correlated (or δ-correlated, white-noise) random forcing as follows:

(4) $$\langle \xi \left(t\right)\xi \left(t^{\prime }\right)\rangle =2D\delta \left(t-t^{\prime }\right),$$

where the angular brackets represent the average over ξ, 〈ξ〉=0, and *D* is the strength of the forcing. The parameter γ is a positive constant. *n* is the order of nonlinearity, which we take to be an odd integer to make x=0 an attractor, and also preserve a reflectional symmetry (under x→-x) of the system.

We note that there are two possible interpretations of Equation (3). Specifically, consider the linear case where $\frac{\partial V}{\partial x}\propto x$. The first interpretation is to view *x* as a velocity, in which case the force term would give a frictional force (e.g., Equation (1.2) in [34]) as $\frac{dv}{dt}=-\gamma v+\xi$, with γ as a frictional (damping) constant. The second interpretation is to take the overdamped limit of the coupled equations for *x* and *v* by dropping $d^{2}x/dt^{2}$ (e.g., Equation (3.130) in [34]) to obtain one equation for *x* from the first line in Equation (3.130). In this case, *x* represents the position and $\gamma^{-1}$ would be the frictional constant, and a harmonic potential would correspond to n=1.

For a deterministic system with ξ=0, the solution to Equation (3) is readily obtained as

(5) $$x\left(t\right)=\frac{x_{0}}{{\left[1+\gamma \left(n-1\right)tx_{0}^{n-1}\right]}^{\frac{1}{n-1}}},$$

where $x_{0}$ is the initial condition x(t=0).

The corresponding Fokker–Planck (FP) equation is [34]

(6) $$\partial_{t}p\left(x,t\right)=\partial_{x}\left(\gamma x^{n}p\right)+D\partial_{xx}p.$$

We first discuss some analytic limiting cases in Section 3, and then present numerical solutions in Section 4.

## 3. Analytic Solutions

### 3.1. Exact Solutions for D=0

In the absence of the stochastic noise D=0, the diffusion term in the Fokker–Planck Equation (6) is zero. In this case, the PDF does not have a stationary solution, but continues to change in time due to the linear/nonlinear force. For instance, for n=1, the PDF’s width becomes exponentially narrow in time as the fluctuation (as well as the mean value) decreases exponentially due to γ>0 (see below). To obtain an analytic solution to Equation (6), we use the method of characteristics. Specifically, we rewrite Equation (6) with D=0 in terms of the total derivative along the characteristic as

(7) $$\frac{dp}{dt}\equiv \frac{\partial p}{\partial t}+\frac{dx}{dt}\frac{\partial p}{\partial x}=n\gamma x^{n-1}p.$$

Here, the characteristic is given by

(8) $$\frac{dx}{dt}=-\gamma x^{n},$$

which is Equation (3) without the stochastic noise ξ. Thus, the characteristic with the initial condition $x\left(t=0\right)=x_{0}$ satisfies Equation (5), which can also be written as

(9) $$x_{0}=\frac{x(t)}{{\left[1-\gamma \left(n-1\right)t{\left[x\left(t\right)\right]}^{n-1}\right]}^{\frac{1}{n-1}}}.$$

Along these characteristics, we rewrite Equation (7) as

(10) $$\frac{dp}{p}=n\gamma x^{n-1}dt=n\gamma \frac{x_{0}^{n-1}dt}{\left[1+\gamma \left(n-1\right)tx_{0}^{n-1}\right]}=\frac{n}{n-1}d\left[\ln\left[1+\gamma \left(n-1\right)tx_{0}^{n-1}\right]\right].$$

The integration of Equation (10) then gives us

(11) $$p\left(x,t\right)=p\left(x_{0},0\right){\left[1+\gamma \left(n-1\right)tx_{0}^{n-1}\right]}^{\frac{n}{n-1}}=p\left(x_{0},0\right){\left[1-\gamma \left(n-1\right)tx^{n-1}\right]}^{-\frac{n}{n-1}}.$$

For simplicity, we consider an initial Gaussian PDF localised around μ as

(12) $$p\left(x_{0},0\right)=\sqrt{\frac{\beta_{0}}{\pi }}e^{-\beta_{0}{\left(x_{0}-\mu \right)}^{2}}.$$

Then, we can find p(x,t) at a later time from Equations (9), (11) and (12) as

(13) $$p\left(x,t\right)=\sqrt{\frac{\beta_{0}}{\pi }}\;\phi^{n}\;e^{-\beta_{0}{\left(x\phi -\mu \right)}^{2}},$$

where

(14) $$\begin{array}{c} \phi ={\left[1-\gamma \left(n-1\right)tx^{n-1}\right]}^{-\frac{1}{n-1}}. \end{array}$$

The maximum amplitude of *p* occurs at that *x* where xϕ-μ=0 in Equation (13), with the value

(15) $$p_{\max}\left(t\right)=\sqrt{\frac{\beta_{0}}{\pi }}\;{\left[1+\gamma \left(n-1\right)t\mu^{n-1}\right]}^{\frac{n}{n-1}}.$$

The linear case n=1 can be obtained by taking the limit n→1 in Equations (13) and (14). One finds that $\phi =e^{\gamma t}$ so that

(16) $$\lim_{n\to 1}p\left(x,t\right)=\sqrt{\frac{\beta }{\pi }}e^{-\beta {\left(x-\mu e^{-\gamma t}\right)}^{2}},$$

where $\beta =\beta_{0}e^{2\gamma t}$. That is, the width of the PDF ($\propto \beta^{-1/2}$) as well as the peak position decrease exponentially. However, the linear case can be solved analytically even when D≠0 [33], so we are here more interested in the nonlinear cases n=3,5,7.

Given p(x,t) in Equation (13), we can further calculate E(t) as follows

(17) $$\begin{array}{ccc} E(t) & = & \gamma^{2}\int dx\;{\left(x\phi \right)}^{2(n-1)}{\left[n-2\beta_{0}x\phi \left(x\phi -\mu \right)\right]}^{2}p\left(x,t\right)\\ & = & \gamma^{2}\int dx_{0}\;x_{0}^{2(n-1)}{\left[n-2\beta_{0}x_{0}\left(x_{0}-\mu \right)\right]}^{2}p\left(x_{0},0\right), \end{array}$$

where we use $x_{0}=x\phi$, $\frac{dx_{0}}{dx}=\phi^{n}$, $p\left(x_{0},0\right)=p\left(x,t\right)\frac{dx}{dx_{0}}$, and $p(x_{0},0)$ is the initial Gaussian PDF in Equation (12). Interestingly, Equation (17) is independent of time, with constant τ. That is, without a stochastic noise, the evolution of PDFs follows a geodesic due to the scaling relation satisfied along the characteristic. For example, we can show that, for n=1, $E=2\gamma^{2}\left(1+\beta_{0}\mu^{2}\right)$ and for n=3, $E=\gamma^{2}\mu^{4}\left[2\beta_{0}\mu^{2}+24+\frac{99}{2\beta_{0}\mu^{2}}+\frac{21}{2{\left(\beta_{0}\mu^{2}\right)}^{2}}\right]$. From these analyses, we can infer that for sufficiently large $\beta_{0}$ such that $\beta_{0}\mu^{n-1}\gg 1$, $E\propto \beta_{0}\mu^{2n}$ to leading order.

### 3.2. Approximate Solutions for D≠0

According to the previous results, the peak amplitudes increase indefinitely in time. The corresponding widths also decrease, and eventually become so narrow that any non-zero *D* will start to play a significant role, and will start to broaden the PDFs again. However, during this phase of the evolution, the PDF width is still smaller than its mean position, so that we can use a quasi-linear analysis to approximate Equation (3) to leading order in $O(x^{\prime }/\langle x\rangle )$ as

(18) $$\frac{dx^{\prime }}{dt}\approx -n\gamma {\langle x\left(t\right)\rangle }^{n-1}x^{\prime }+\xi ,$$

where $x^{\prime }\left(t\right)=x\left(t\right)-\langle x\left(t\right)\rangle$ is the fluctuation, with $\langle x^{\prime }\left(t\right)\rangle =0$. $x^{\prime }$ in Equation (18) satisfies the Ornstein–Uhlenbeck process with the effective damping constant

(19) $$\gamma_{e}\left(t\right)=n\gamma {\langle x\left(t\right)\rangle }^{n-1}.$$

Thus, in this stage, the PDFs remain essentially Gaussian and evolve as

(20) $$p\left(x,t\right)=\sqrt{\frac{\beta }{\pi }}e^{-\beta {\left(x-\langle x\rangle \right)}^{2}},$$

where

(21) $$\begin{array}{ccc} \langle x\rangle & = & \frac{\mu }{{\left[1+\gamma \left(n-1\right)\mu^{n-1}t\right]}^{\frac{1}{n-1}}}, \end{array}$$

(22) $$\begin{array}{ccc} \frac{1}{2\beta } & = & \frac{e^{-2G(t)}}{2\beta_{0}}+\frac{D(1-e^{-2G(t)})}{\gamma_{e}}, \end{array}$$

(23) $$\begin{array}{ccc} G(t) & = & \int_{0}^{t}dt^{\prime }\gamma_{e}\left(t^{\prime }\right)=\frac{n}{n-1}\ln\left[1+\gamma \left(n-1\right)\mu^{n-1}t\right]. \end{array}$$

The term $e^{-2G}/2\beta_{0}$ in Equation (22) represents the narrowing of the initial PDF width as discussed in the previous section. However, note that, when $1/2\beta_{0}\ll D/\gamma_{e}$, a PDF can maintain the same width set by the constant value $D/\gamma_{e}$ at the initial stage; that is, there is no nondiffusive evolution phase. See [33] for such a case with n=3. For the $1/2\beta_{0}>D/\gamma_{e}$ case, we consider here, the transition from the nondiffusive evolution phase to the quasi-linear Gaussian evolution phase occurs when $e^{-2G}/2\beta_{0}$ becomes comparable to $D/\gamma_{e}$, leading to the following criterion for the first transition timescale $t_{1}$:

(24) $$\begin{array}{c} 2G∼\ln\frac{\gamma_{e}}{2\beta_{0}D}∼\ln\frac{n\gamma {\langle x\rangle }^{n-1}}{2\beta_{0}D}, \end{array}$$

using $\gamma_{e}=n\gamma {\langle x\rangle }^{n-1}$. By using Equation (23) and $\langle x\rangle =\mu /{\left[1+\gamma \left(n-1\right)\mu^{n-1}t_{1}\right]}^{\frac{1}{n-1}}$, we obtain

(25) $${\left[1+\gamma \left(n-1\right)\mu^{n-1}t_{1}\right]}^{\frac{3n-1}{n-1}}∼\frac{\gamma n}{2\beta_{0}D}\mu^{n-1}.$$

For large time $\gamma \left(n-1\right)\mu^{n-1}t_{1}\gg 1$, Equation (25) thus yields

(26) $$t_{1}\propto D^{-\frac{n-1}{3n-1}}\;\mu^{-\frac{2n(n-1)}{3n-1}}.$$

Note in particular how both exponents are negative for n=3,5,7, so that smaller *D* and/or μ yield a larger $t_{1}$. Next, for D≠0 and $\gamma \left(n-1\right)\mu^{n-1}t\gg 1$, we approximate Equation (22) as

(27) $$\frac{1}{2\beta }∼\frac{D}{\gamma_{e}}∼\frac{n-1}{n}Dt,$$

using also $\gamma_{e}\propto t^{-1}$. Thus, the PDF width increases as ${\left(Dt\right)}^{1/2}$, while the maximum amplitude decreases as

(28) $$p_{\max}\left(t\right)\propto \beta^{1/2}\propto {\left(Dt\right)}^{-1/2},$$

typical of Brownian motion [34]. Using Equation (26) in either Equation (15) or (28)—$t_{1}$ after all is precisely the time when one scaling transitions to the other, so they should agree at that time—we obtain the scalings of the overall maximum amplitude $p_{\mathrm{Max}}$ reached throughout the entire evolution as

(29) $$p_{\mathrm{Max}}\propto D^{-\frac{n}{3n-1}}\;\mu^{\frac{n(n-1)}{3n-1}}.$$

### 3.3. Final Stationary Distribution

For even greater times, fluctuations become stronger while the mean values decrease. The quasi-linear analysis in the previous section is therefore eventually no longer applicable, and numerical solutions are crucial. The final stationary solution of Equation (6) can however be computed analytically from $D\partial_{x}p=-\gamma x^{n}p$ and becomes

(30) $$p\left(x\right)={\left(\frac{\gamma }{D(n+1)}\right)}^{1/(n+1)}\frac{n+1}{\Gamma (1/(n+1))}\;\exp\left(-\frac{\gamma }{D(n+1)}\;x^{n+1}\right).$$

Thus, the maximum amplitude

(31) $$p_{\max}\propto D^{-\frac{1}{n+1}},$$

while the width of the PDF is proportional to $D^{\frac{1}{n+1}}$. Finally, the transition from the intermediate stage in the previous section to this final stage occurs when the two formulas for the widths ${\left(Dt\right)}^{1/2}$ and $D^{\frac{1}{n+1}}$ become comparable, yielding the second transition timescale

(32) $$t_{2}\propto D^{-\frac{n-1}{n+1}}.$$

In the following section, we see how numerical solutions compare with some of these predictions such as the two timescales $t_{1}$ and $t_{2}$, as well as explore other aspects of the solutions for which no analytic predictions are possible.

## 4. Numerical Solutions

For D≠0, an exact analytic solution to the Fokker–Planck Equation (6) only exists for the linear case n=1 [33]. For the nonlinear cases n=3,5,7 that are the focus of this paper, we must resort to numerical solutions. Without loss of generality, we set γ=1. The interval in *x* is fixed to be [-1,1], rather than the original [-∞,∞]. This may seem drastic, but actually involves no real loss of generality either, since any finite interval can always be mapped to [-1,1] by suitably rescaling *x*, *t* and *D*. As long as the initial condition (and *D*) are chosen such that *p* would be negligible outside [-1,1] anyway, solving the FP equation only on [-1,1], and with p=0 boundary conditions is then equivalent in all essentials to the original infinite interval.

The numerical solution is done by second-order finite-differencing in *x*, with up to $M=2\times 10^{6}$ grid points. The time-stepping is also second-order accurate, with increments as small as $\Delta t=10^{-6}$. Both *M* and Δt were varied to check the accuracy of the solutions. In the later stages, when the PDFs are evolving to increasingly broad profiles, *M* can also be decreased, and Δt increased, while still preserving accuracy. Regridding the solutions in this way is indeed crucial, since the final adjustment timescale $t_{2}$ is extremely long for small *D*, and could not be reached if *M* and Δt were kept fixed at their initial values.

In [33], we considered the initial condition

(33) $$p=\frac{1}{\sqrt{\pi \;10^{-8}}}\exp\left[-\frac{{\left(x-0.7\right)}^{2}}{10^{-8}}\right],$$

and then compared numerical solutions for n=3 and $D=10^{-3}$ to $10^{-7}$ with the corresponding analytical solutions for n=1. That is, the initial peak was extremely narrow, and diffusion was greater than what we consider here. As a result of these choices, the peaks only became broader but never narrower; the nondiffusive regime in Section 3.1 simply does not exist in this case.

In contrast, in this work, we take the initial condition

(34) $$p=\frac{1}{\sqrt{2\pi \;10^{-6}}}\exp\left[-\frac{{\left(x-\mu \right)}^{2}}{2\times 10^{-6}}\right],$$

and $D=10^{-6}$ to $10^{-9}$. By starting with broader peaks and having smaller *D*, we do have an initial nondiffusive regime here, and are able to observe the narrowing of the peaks predicted by Equations (13) and (14). We begin by fixing the initial peak position μ=0.65; below, we also consider the range μ=[0.01,0.75].

**Figure 1:**
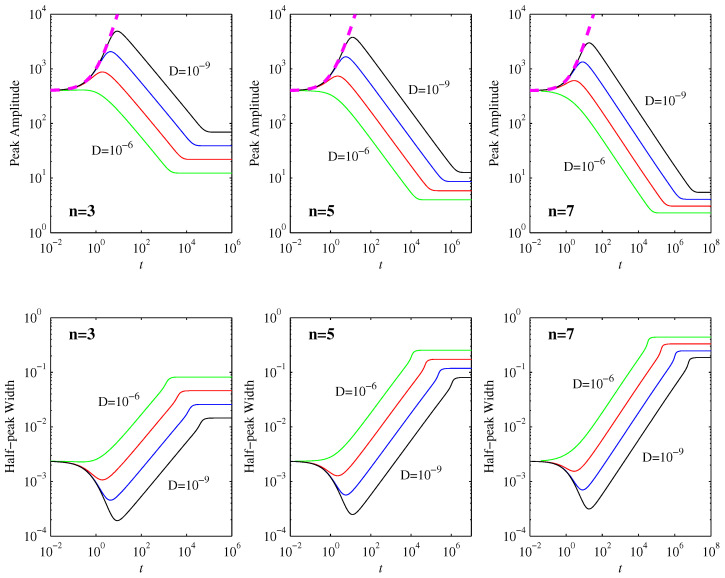
The top row shows the peak amplitudes as functions of time, for the initial condition in Equation (34) with μ=0.65, and $D=10^{-6}$ to $10^{-9}$ as indicated. The three panels show n=3,5,7, as labeled. The thick dashed (magenta) lines correspond to the analytic result (Equation (15)) that applies in the nondiffusive phase. The bottom row shows the equivalent widths at half-peak, which are inversely proportional to the peak amplitudes. The standard deviation ${\langle {\left(x-\langle x\rangle \right)}^{2}\rangle }^{1/2}$ follows exactly the same pattern as the half-peak widths.

Figure 1 shows how the peak amplitudes evolve in time. We can clearly see the three regimes deduced in Section 3: the peaks initially increase, in excellent agreement with Equation (15), then they decrease in agreement with Equation (28), and finally they equilibrate to Equation (31). To more quantitatively compare the overall peak amplitudes $p_{\mathrm{Max}}$ and the corresponding times $t_{\mathrm{Max}}$ at which they occur with the analytic predictions given by Equations (26) and (29), we note first that $D=10^{-6}$ is clearly not yet sufficiently small for there to be an initial nondiffusive regime at all (for this particular width of the initial condition). Even $D=10^{-7}$ only follows the nondiffusive Equation (15) for a very brief time, not yet long enough to be in the $\gamma \left(n-1\right)\mu^{n-1}t\gg 1$ regime where Equations (26) and (29) are expected to apply. Table 1 therefore compares the $D=10^{-8}$ and $10^{-9}$ cases, and uses them to extract scaling exponents of the form $D^{-\alpha }$. We see that the agreement of $p_{\mathrm{Max}}$ with Equation (29) is virtually perfect. The corresponding times $t_{\mathrm{Max}}$ are close to the expected scaling $t_{1}$, but are not fully in the asymptotic limit yet. This is hardly surprising, since even for $D=10^{-9}$ the $t_{\mathrm{Max}}$ values in Table 1 only have $\gamma \left(n-1\right)\mu^{n-1}t_{\mathrm{Max}}∼8$ (for all three *n* values).

To similarly test the scalings that are predicted for μ, further runs were done with fixed $D=10^{-9}$, and μ=0.55 and 0.75. As Table 2 shows, the extracted exponents are again in very good ($t_{\mathrm{Max}}$) and perfect ($p_{\mathrm{Max}}$) agreement with the predicted asymptotic scalings. The interesting feature that larger initial positions μ have smaller times to reach the maximum peak amplitude is certainly very well reproduced. Note finally that the two μ values used to extract these exponents in Table 2 differ by less than a factor of two even, and would thus certainly not be enough to extract reliable scaling exponents if we did not already have robust analytic predictions. As we also show in more detail below, in principle, it would be possible to have the analytic predictions extend over an arbitrarily large range in μ, but that would require increasingly small *D* as well, which becomes numerically too time-consuming.

**Figure 2:**
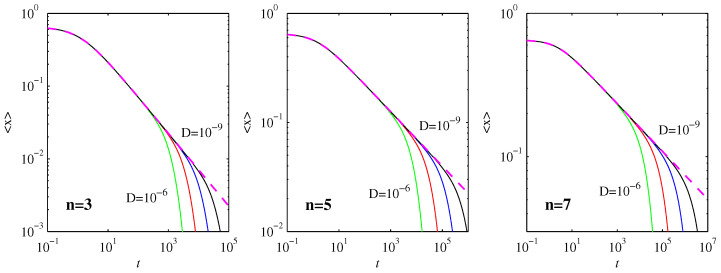
The mean values 〈x〉 as functions of time, for the solutions in Figure 1. The dashed (magenta) lines show Equation (5) that applies in the nondiffusive case.

Returning to the μ=0.65 runs in Figure 1 and Figure 2 shows the expectation values 〈x〉=∫xpdx, and how they compare with the nondiffusive result (Equation (5)). The agreement is close to perfect even after the first timescale $t_{1}$ is reached. It is only once the very long second timescale $t_{2}$ is reached that 〈x〉 approaches 0 exponentially, far more rapidly than the $t^{-\frac{1}{n-1}}$ power law scaling in (5).

**Figure 3:**
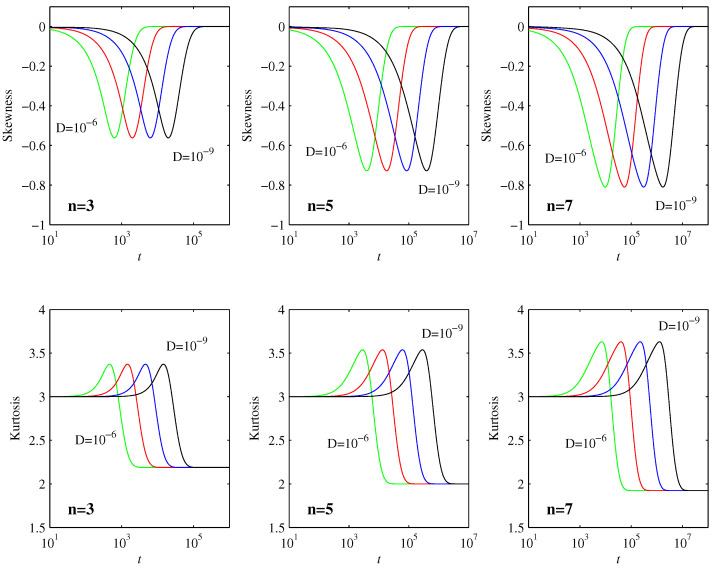
The top row shows the skewness $\int {\left[\left(x-\langle x\rangle \right)/\sigma \right]}^{3}\;p\;dx$, and the bottom row shows the kurtosis $\int {\left[\left(x-\langle x\rangle \right)/\sigma \right]}^{4}\;p\;dx$, as functions of time. The labeling of *D* and *n* is as in Figure 1 and Figure 2. The peaks in both quantities occur at times in essentially perfect agreement with $t_{2}$ in (32).

Figure 3 shows two commonly used diagnostic quantities, the skewness $\int {\left[\left(x-\langle x\rangle \right)/\sigma \right]}^{3}\;p\;dx$ and the kurtosis $\int {\left[\left(x-\langle x\rangle \right)/\sigma \right]}^{4}\;p\;dx$, where $\sigma ={\left[\int {\left(x-\langle x\rangle \right)}^{2}\;p\;dx\right]}^{1/2}$ is the standard deviation. Skewness is a measure of how asymmetric a PDF is about its peak, with a value of 0 indicating a symmetric peak. Kurtosis measures the flatness of a PDF, especially in comparison with a Gaussian, which has kurtosis=3. We see that skewness≈0 and kurtosis≈3 is maintained all the way until the final equilibration timescale $t_{2}$ is reached, indicating that the PDFs remain largely Gaussian up to this time, as predicted in Section 3.2. For the final equilibrated profiles in Equation (30), the skewness is again 0, whereas the kurtosis has values that depend on *n* (the precise values can be evaluated analytically in terms of Γ functions, but are not particularly insightful). It is interesting though to note in Figure 3 that both skewness and kurtosis exhibit non-monotonic behavior during the final adjustment process, where the PDFs transition from being largely Gaussian to their final form. Note also how the maxima reached during this transition process are independent of *D*, and even broadly similar for the different values of *n*.

**Figure 4:**
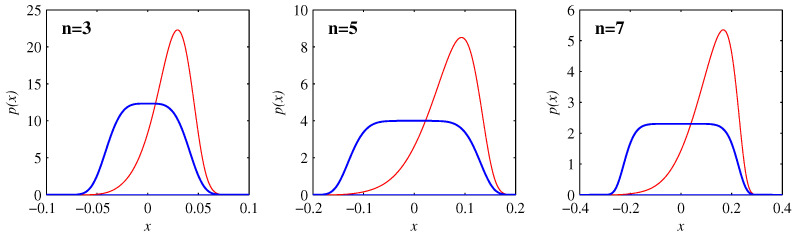
The heavy (blue) lines show the equilibrium profiles in Equation (30), for $D=10^{-6}$, and n=3,5,7 as indicated. The lighter (red) lines show p(x) at the t=629,3930,9794, for n=3,5,7, respectively. As shown in Figure 3, these are the times when the skewness reaches its maximum negative values. Results for other values of *D* are identical, once *x* and p(x) are rescaled as in Equation (31), and *t* is shifted as in Figure 3 to consistently have the correct skewness values.

Figure 4 compares the PDFs at the times when the skewness reaches its maximum (negative) value with the final equilibrated profiles in Equation (30). The entire evolution is then clear: as long as the PDFs are well outside their final profiles, they remain essentially Gaussian, with widths as in Figure 1 and positions as in Figure 2. The skewness and kurtosis start to deviate significantly from their Gaussian values when the PDFs start to reach their final positions, with the maximum skewness as in Figure 4. The final rearrangement for $t>t_{2}$ then merely adjusts to the final profiles (30), but with relatively little further movement of the PDFs.

**Figure 5:**
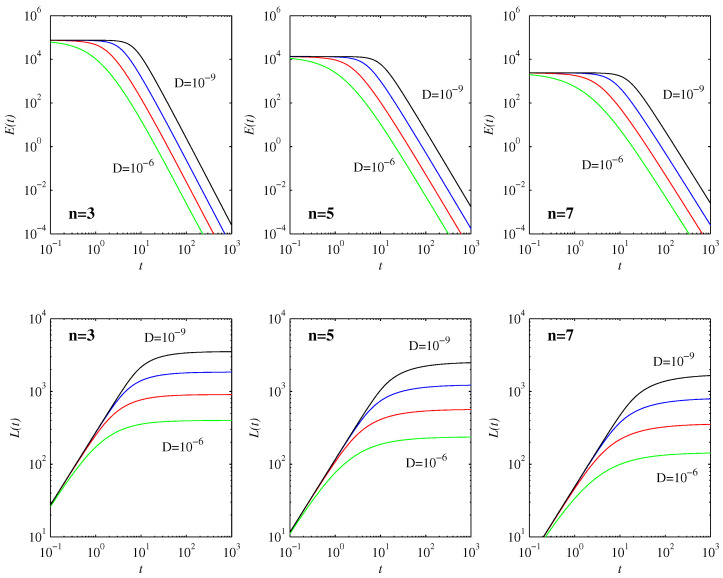
The top row shows E(t) and the bottom row shows L(t). The labeling of *D* and *n* is as in Figure 1, Figure 2 and Figure 3. The peaks are initially located at μ=0.65, consistent with the initial plateau in E being reduced by a factor of $0.65^{4}=0.1785$ when comparing n=3 and 5, and similarly n=5 and 7.

Figure 5 shows the diagnostic quantities E(t) and L(t). As predicted by Equation (17), E remains constant in the initial nondiffusive phase. Once the first timescale $t_{1}$ is reached, E decreases as $t^{-\frac{3n-1}{n-1}}D^{-1}$. Once the second timescale $t_{2}$ is reached, E decreases exponentially. (This is not included in Figure 5, however, as $E\propto D^{\frac{2(n-1)}{n+1}}$ at that point, and is thus already negligibly small.) The behavior of L is as expected: while E is constant, L increases linearly in time, and, once E starts decreasing, L levels off to its final value $L_{\infty }$.

Based on the scaling for E, namely that $E\propto \mu^{2n}$ initially, and the range $t_{1}$ for which this applies, we expect at least a lowest-order estimate for $L_{\infty }$ to be given simply by

(35) $$L_{\infty }\propto \sqrt{E}\;t_{1}\propto D^{-\frac{n-1}{3n-1}}\;\mu^{\frac{n(n+1)}{3n-1}}.$$

**Figure 6:**
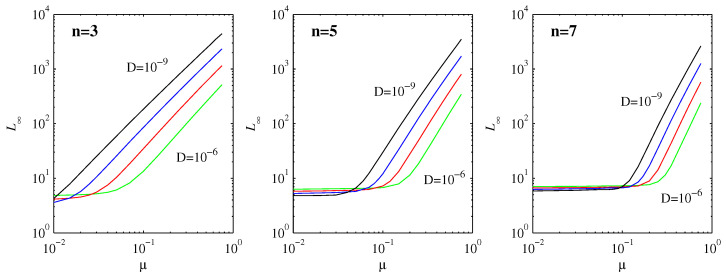
$L_{\infty }$ as a function of the initial position μ, for $D=10^{-6}$ to $10^{-9}$ and n=3,5,7 as labeled.

Figure 6 shows numerically computed results, for the same $D=10^{-6}$ to $10^{-9}$ range considered throughout, and μ=[0.01,0.75]. At least for sufficiently large μ, we do indeed see clear evidence of power-law behavior, with a negative exponent for *D* and a positive one for μ. Concentrating on the variation with μ, for n=3, the slopes vary between 1.8 for $D=10^{-6}$ and 1.6 for $D=10^{-9}$; for n=5, they vary between 2.7 and 2.3; and for n=7, they vary between 3.5 and 2.9. By comparison, according to Equation (35), the exponent should be $\frac{n(n+1)}{3n-1}$, which yields 1.5, 2.1, 2.8 for n=3,5,7, respectively. The agreement is thus quite good, especially when we recall (Table 1 and Table 2) that even $D=10^{-9}$ was not quite small enough yet to obtain precise agreement with the asymptotic formula for $t_{1}$, which in turn affects Equation (35) as well.

$L_{\infty }$ as a function of μ provides a mapping from the physical distance μ (the distance between the peak position μ of an initial PDF and the final equilibrium point 0) to the information length (the total number of statistically different states of a system reaching a final stationary PDF from an initial PDF). From Equation (35), this mapping is linear for a linear force; that is, $L_{\infty }$ is linearly proportional to the physical distance. This linear relation breaks down for nonlinear forces as $L_{\infty }$ depends nonlinearly on the physical distance μ. Specifically, for n=3, $L_{\infty }\propto \mu^{1.5}$; for n=5, $L_{\infty }\propto \mu^{2.1}$; and, for n=7, $L_{\infty }\propto \mu^{2.8}$. Thus, we can envision that nonlinear forces affect the information geometry, changing a linear (flat) coordinate to a power-law (curved) coordinate. This is reminiscent of gravity changing a flat to a curved space-time. Furthermore, interestingly, Equation (35) shows that $L_{\infty }$ is independent of *D* for the linear force, manifesting the information geometry as independent of the resolution (set by *D*). In contrast, $L_{\infty }$ decreases with n=3,5,7 as $D^{-1/4},\;D^{-2/7},\;D^{-3/10}$, suggesting that the information geometry is fractal, depending on the resolution. This would be equivalent to the *I* theorem of [14].

Finally, what about the small μ limit in Figure 6, which clearly does not follow the expected scaling (35)? The explanation is that the initial position μ is already within the $O(D^{\frac{1}{n+1}})$ width of the final distribution in Equation (30). In this limit, the behavior is different, and the peak merely spreads out, resulting in a small and relatively uniform $L_{\infty }$. For any given μ, *D* must therefore satisfy $D\ll \mu^{n+1}$ to be in the regime where Equation (35) is expected to apply. That is, in principle, it would be possible to have Equation (35) apply over several orders of magnitude in μ, but *D* would have to be far smaller than is numerically feasible.

## 5. Conclusions

The motivation of this research was to elucidate the link between force and geometry in a stochastic system. To this end, we investigated the transient relaxation of initial PDFs of a stochastic variable *x* under different nonlinear forces proportional to $-x^{n}$ (n=3,5,7) and different strength *D* of δ-correlated stochastic noise. We identified the three main stages consisting of nondiffusive evolution, quasi-linear Gaussian evolution and settling into stationary PDFs. The strength of the stochastic noise is shown to play a crucial role in determining these timescales $t_{1}\propto D^{-\frac{n-1}{3n-1}}$ and $t_{2}\propto D^{-\frac{n-1}{n+1}}$, as well as the peak amplitude and width of PDFs. Note in particular how both timescales have (*n*-dependent) power-law scalings with *D*, in sharp contrast with the linear n=1 case, where the entire evolution occurs on t=O(1) timescales, with no dependence on *D*.

We further computed the rate of information change, and the information length L(t) representing the number of distinguishable statistical states that the system evolves through in time. We identified a robust geodesic (where the information changes at a constant rate) in the initial nondiffusive stage, and mapped out a geometric structure of an attractor as $L_{\infty }\propto \mu^{m}$, where μ is the peak position of the initial Gaussian PDF. For sufficiently small *D* the exponent *m* was shown to be $\frac{n(n+1)}{3n-1}$, but still exhibits (moderate) variation with *D* even for values as small as $10^{-9}$. This suggests that the geometry is curved by the nonlinear interaction, in contrast with the linear geometry of the Ornstein-Uhlenbeck process which has m=1 and no variation with *D*. Our results thus highlight ubiquitous power-laws and multi-scalings of information geometry.

This work will form a basis for future investigation of a more general case of the superposition of nonlinear forces with different *n*s. Multiplicative noise would also be interesting, but the situation becomes somewhat more complicated as a deterministic force could appear as $-x^{n}$ as here, but the noise ξ could appear as $\xi x^{m}$, with *m* not necessarily the same as *n*. Fractional noise would also be worth pursuing, although this would be challenging both analytically and numerically. Finally, it will also be interesting to extend our work to analyse a heterogeneous, linear system [35,36,37], and to explore applications of our work to estimators.

## Figures and Tables

**Table 1:** The first two rows show the overall peak amplitudes $p_{\mathrm{Max}}$ and the times $t_{\mathrm{Max}}$ at which they occur, for the results in Figure 1. $D=10^{-8}$ and $10^{-9}$, and n=3,5,7 as indicated, and all results at μ=0.65. The row labeled “Exponent” uses the ratios of the two *D* values to extract scaling exponents of the form $D^{-\alpha }$. The final row compares these numerically deduced exponents with the analytic predictions from Equations (26) and (29). That is, the exponent α should equal $\frac{n-1}{3n-1}$ for $t_{\mathrm{Max}}$, and $\frac{n}{3n-1}$ for $p_{\mathrm{Max}}$.

	n=3		n=5		n=7	
	$t_{\mathrm{Max}}\;$	$p_{\mathrm{Max}}$	$t_{\mathrm{Max}}\;$	$p_{\mathrm{Max}}$	$t_{\mathrm{Max}}\;$	$p_{\mathrm{Max}}$
$D=10^{-8}$	4.42	2063	5.82	1661	8.17	1337
$D=10^{-9}$	8.80	4888	12.55	3776	18.58	2998
Exponent	0.30	0.375	0.33	0.357	0.36	0.349
(26), (29)	0.25	0.375	0.29	0.357	0.30	0.350

**Table 2:** The first two rows are as in Table 1, but now for fixed $D=10^{-9}$, μ=0.55 and 0.75, and n=3,5,7 as indicated. The row labeled “Exponent” uses the ratios of the two μ values to extract scaling exponents of the form $\mu^{\delta }$. The final row compares these numerically deduced exponents with the analytic predictions from Equations (26) and (29). That is, the exponent δ should equal $-\frac{2n(n-1)}{3n-1}$ for $t_{\mathrm{Max}}$, and $\frac{n(n-1)}{3n-1}$ for $p_{\mathrm{Max}}$.

	n=3		n=5		n=7	
	$t_{\mathrm{Max}}\;$	$p_{\mathrm{Max}}$	$t_{\mathrm{Max}}\;$	$p_{\mathrm{Max}}$	$t_{\mathrm{Max}}\;$	$p_{\mathrm{Max}}$
μ=0.55	11.17	4314	19.74	2975	35.87	2105
μ=0.75	7.18	5439	8.48	4631	10.46	4035
Exponent	-1.4	0.75	-2.7	1.43	-4.0	2.10
(26), (29)	-1.5	0.75	-2.9	1.43	-4.2	2.10
